# MiR-590-5p Inhibits Oxidized- LDL Induced Angiogenesis by Targeting LOX-1

**DOI:** 10.1038/srep22607

**Published:** 2016-03-02

**Authors:** Yao Dai, Zhigao Zhang, Yongxiang Cao, Jawahar L. Mehta, Jun Li

**Affiliations:** 1 School of Pharmacy, Anhui Medical University, Hefei, Anhui, 230032 People’s Republic of China.; 2 Department of Medicine, Central Arkansas Veterans Healthcare System and the University of Arkansas for Medical Sciences, Little Rock, AR 72205; 3 Department of Internal Medicine, The First Affiliated Hospital of Anhui Medical University, Hefei, Anhui, 230022 People’s Republic of China

## Abstract

Oxidized low-density lipoprotein (ox-LDL) is, at least in part, responsible for angiogenesis in atherosclerotic regions. This effect of ox-LDL has been shown to be mediated through a specific receptor LOX-1. Here we describe the effect of miR-590-5p on ox-LDL-mediated angiogenesis in *in vitro* and *in vivo* settings. Human umbilical vein endothelial cells (HUVECs) were transfected with miR-590-5p mimic or inhibitor followed by treatment with ox-LDL. In other experiments, Marigel plugs were inserted in the mice subcutaneous space. Both *in vitro* and *in vivo* studies showed that miR-590-5p mimic (100 nM) inhibited the ox-LDL-mediated angiogenesis (capillary tube formation, cell proliferation and migration as well as pro-angiogenic signals- ROS, MAPKs, pro-inflammatory cytokines and adhesion-related proteins). Of note, miR-590-5p inhibitor (200 nM) had the opposite effects. The inhibitory effect of miR-590-5p on angiogenesis was mediated by inhibition of LOX-1 at translational level. The inhibition of LOX-1 by miR-590-5p was confirmed by luciferase assay. In conclusion, we show that MiR-590-5p inhibits angiogenesis by targeting LOX-1 and suppressing redox-sensitive signals.

Angiogenesis in the arterial wall promotes atherogenesis and is a key process in rupture-prone atherosclerotic lesions^1^. Oxidized low density lipoprotein (ox-LDL) stimulates reactive oxygen species (ROS) formation followed by release of vascular endothelial growth factor (VEGF) in endothelial cells, which stimulates angiogenesis. Redox-sensitive pathways, such as activation of mitogen-activated protein kinases (MAPKs), have been shown to be involved in angiogenesis^2^. However, the underlying mechanism of this process has not been fully elucidated. Thus, few efficient anti-angiogenic therapies have been designed to treat angiogenesis in atherosclerosis^3^.

MicroRNAs (miRs) are a group of small non-coding RNAs, which regulate almost all physiological and pathological processes in mammals, through degrading target mRNAs or inhibiting their translation^4^. Growing body of evidence has shown that miRNAs are involved in the regulation of angiogenesis seen in certain cancers and their metastatic spread^5^.

Lectin-type oxidized LDL receptor 1 (LOX-1) is a receptor protein which binds, internalizes and degrades ox-LDL. Our recent observations in cultured human umbilical vein endothelial cells (HUVECs) showed a sustained decrease in miR-590-5p expression, along with an increase in LOX-1 expression, particularly when cells were treated with ox-LDL. Further, the LOX-1 3′-untranslated region (UTR) was predicted to be a putative target site for miR-590-5p, using bioinformatics tools Miranda, Pic Tar and Targetscan 6.0^6^,^7^. These observations initiated our hypothesis: miR-590-5p inhibits ox-LDL-mediated angiogenesis through LOX-1/redox-sensitive and MAPK pathways in the formation of atherosclerosis.

## Results

### LOX-1, VEGF and miR-590-5p expression and angiogenesis in response to ox-LDL

**Figure 1:**
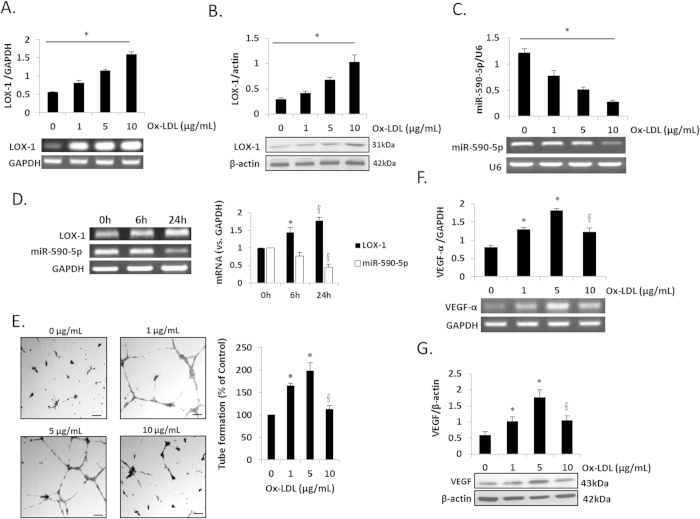
LOX-1, VEGF, miR-590-5p expression and capillary tube formation in HUVECs treated with ox-LDL. (**A,B**) LOX-1 (mRNA and protein) expression in HUVECs treated with ox-LDL for 24 h; (**C**) miR-590-5p expression in response to ox-LDL; (**D**) Different time points after ox-LDL treatment; (**E**) Capillary tube formation in HUVECs treated with ox-LDL, Scale bar = 20 μm; (**F,G**) VEGF (mRNA and protein) in HUVECs treated with ox-LDL. *P < 0.05 vs. ox-LDL 0 μg/mL (Control); ^§^P < 0.05 vs. ox-LDL 5 μg/mL; For Fig. 1D, *P < 0.05 vs. ox-LDL 0 h (Control); ^§^P < 0.05 vs. ox-LDL 6 h, (ox-LDL, 5 μg/mL); LOX-1, Lectin-like oxidized low-density lipoprotein scavenger receptor-1; ox-LDL, oxidized low density lipoprotein; VEGF, vascular endothelial growth factor. Data based on six independent experiments.

We measured LOX-1, VEGF and miR-590-5p expression in HUVECs treated with different concentrations of ox-LDL to select an appropriate concentration for subsequent experiments. As shown in Fig. 1A–C, ox-LDL treatment of HUVECs resulted in a concentration-dependent increase in LOX-1 (mRNA and protein). Simultaneously miR-590-5p expression was noted to decrease (P < 0.05 vs. Control). Changes in LOX-1 and miR-590-5p expression were time-dependent and peaked at 24 h after ox-LDL treatment (Fig. 1D).

As shown earlier, small concentrations of ox-LDL (1–5 μg/mL) augmented tube formation (angiogenesis) and VEGF expression (mRNA and protein) (Fig. 1E–G) (P < 0.05 vs. Control). Treatment of HUVECs with larger concentration of ox-LDL (10 μg/mL) resulted in a decrease in capillary tube formation and VEGF expression (P < 0.05 vs. ox-LDL 5 μg/mL). Hence, we used 5 μg/mL concentration of ox-LDL in all subsequent experiments.

### miR-590-5p and LOX-1 expression

**Figure 2:**
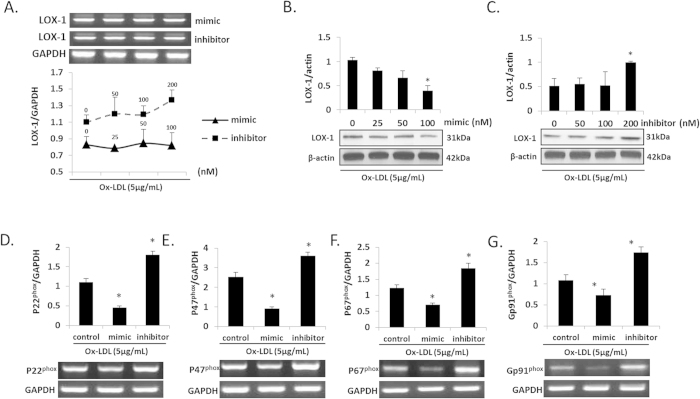
Expression of LOX-1 and NADPH oxidases in HUVECs transfected with miR-590-5p mimic or its inhibitor. (**A–C**) LOX-1 expression (protein and mRNA) in HUVECs treated with different concentration of miR-590-5p mimic or its inhibitor; (**D–G**) NADPH oxidases (mRNA) in HUVECs treated with different concentration of miR-590-5p mimic or its inhibitor (+ox-LDL); *P < 0.05 vs. miR-590-5p mimic or inhibitor (0 nM); Abbreviations as in previous figure.

We selected a range of concentrations to check the most effective concentration of miR-590-5p for our experiments. As shown in Fig. 2A–C, the mRNA expression of LOX-1 was unchanged whether the HUVECs were treated with the miRNA mimic or inhibitor. The mimic had no effect at <100 nM concentration, and the inhibitor had no effect at concentration <200 nM. Transfection of cells with miR-590-5p mimic (100 nM) (transfection efficiency ≈ 75%) significantly decreased LOX-1 protein expression while transfection with miR-590-5p inhibitor (200 nM) (transfection efficiency ≈ 68%) significantly increased it (P < 0.05, miR-590-5p mimic or the inhibitor vs. Control). Compared to negative control, LOX-1 expression was significantly influenced by miR-590-5p mimics and inhibitors. (See Supplementary Fig. S1).

### miR-590-5p and angiogenesis and oxidative stress

**Figure 3:**
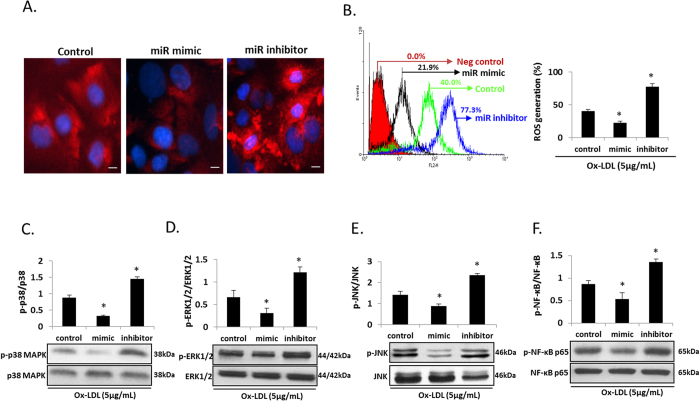
Mitochondrial ROS generation and pro-inflammatory factors in HUVECs transfected with miR-590-5p mimic or its inhibitor. (**A,B**) Immunofluorescence (20×) and flow cytometry measurements for mtROS generation; Scale bar = 20 μm; (**C–F**) Phosphorylation of p38 MAPK, ERK1/2, JNK and NF-κB p65 proteins; *P < 0.05 vs. Control; ROS, reactive oxygen species; p38 MAPK, p38 mitogen-activated protein kinase; ERK1/2, extracellular-signal-regulated kinases 1/2; JNK, c-Jun N-terminal kinase; NF-κB p65, nuclear factor kappa B p65. Data based on six independent experiments.

Since LOX-1 activation leads to the activation of NADPH oxidases, mtROS generation and release of pro-inflammatory cytokines, we checked as to how miR-590-5p may modulate these phenomena in HUVECs. As shown in Fig. 2D-G, mRNA expression of NADPH oxidases (subunits p22^phox^, p47^phox^, p67^phox^ and Gp91^phox^) decreased in HUVECs transfected with the miR-590-5p mimic, and it increased in HUVECs transfected with the miR-590-5p inhibitor (both P < 0.05 vs. Control). Mitochondrial ROS production showed a similar pattern as NADPH oxidases gene expression (indicated by immunofluorescence and by flow cytometry) (P < 0.05 vs. Control) (Fig. 3A,B).

### miR-590-5p and pro-inflammatory cytokines

As shown in Fig. 3C–F, the levels of phosphorylated p38 MAPK, ERK1/2, JNK and NF-κB p65 decreased in HUVECs in response to ox-LDL when the cells had been previously transfected with miR-590-5p mimic, and the levels increased when the cells had been previously transfected with miR-590-5p inhibitor (both P < 0.05 vs. Control). Total protein expression of p38 MAPK, ERK1/2, JNK and NF-κB p65 remained unchanged whether cells were transfected with the mimic or the inhibitor.

### miR-590-5p and angiogenesis- related signaling

**Figure 4:**
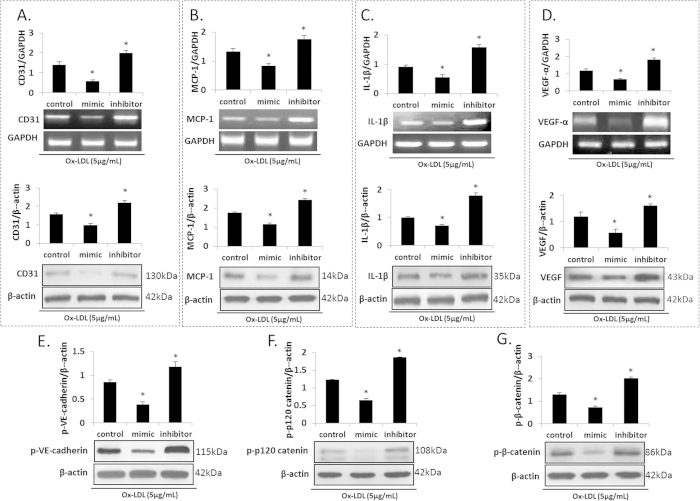
Angiogenesis and related factors in HUVECs transfected with miR-590-5p mimic or its inhibitor. (**A–D**) Expression (mRNA and protein) of CD31, MCP-1, IL-1β, and VEGF; (**E–G**) Expression of adhesion proteins -VE-cadherin, p-p120 catenin and p-β-catenin; *P < 0.05 vs. Control; MCP-1, Monocyte chemoattractant protein-1; IL-1β, interleukin-1 beta. Data based on six independent experiments.

To further confirm the effect of miR-590-5p on angiogenesis, we checked some important angiogenic signals. As shown in Fig. 4A–D, the expression (mRNA and protein) of CD31, MCP-1, IL-1β and VEGF decreased in the miR-590-5p mimic group and increased in the miR-590-5p inhibitor group (both P < 0.05 vs. Control). The phosphorylation of adhesion-related proteins, such as VE-cadherin, p120 catenin and β-catenin, was downregulated in the miR-590-5p mimic and upregulated in the miR-590-5p inhibitor group (both P < 0.05 vs. Control) (Fig. 4E–G).

### miR-590-5p and capillary tube formation

**Figure 5:**
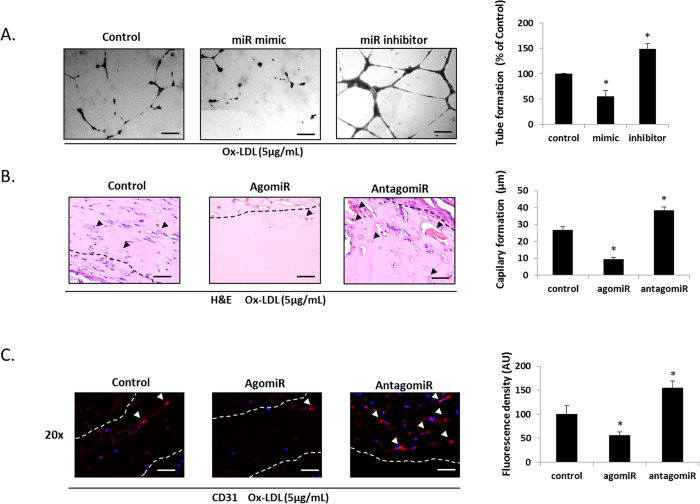
Capillary tube formation, cell migration and proliferation and the role of miR-590-5p. (**A**) Tube formation from HUVECS treated with miR-590-5p mimic or its inhibitor (+ox-LDL), (**B,C**) the effect of agomiR or antagomiR (H&E staining and CD31) on capillary formation in the mouse models (blood vessels indicated with arrowheads, or CD31 (red) and DAPI (blue); Dotted lines indicate tissue and Matrigel border) *P < 0.05 vs. Control. Scale bar = 20 μm. Data based on six independent experiments.

Capillary tube formation from HUVECs decreased in the miR-590-5p mimic group and increased in the miR-590-5p inhibitor group (P < 0.05 vs. Control, Fig. 5A). Sections of matrigel plugs from the mouse model stained with H&E and CD31 antibody in also showed the same pattern (Fig. 5B,C).

### miR-590-5p and cell migration and proliferation

**Figure 6:**
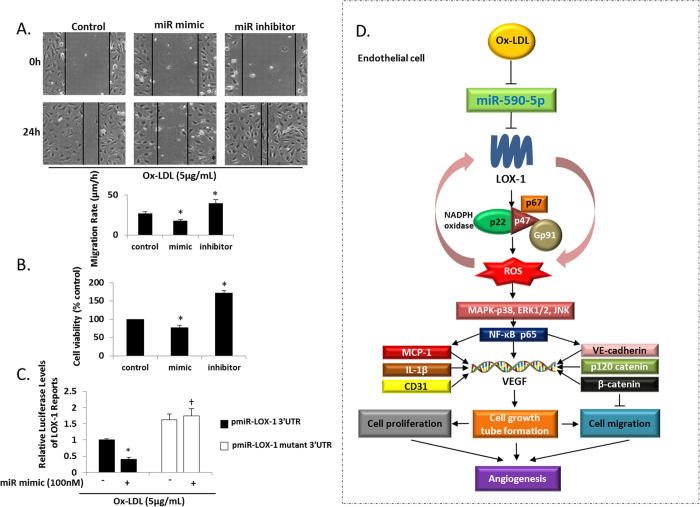
(**A**) Effect of miR-590-5p mimic or its inhibitor on the wound healing assay; (**B**) MTT assay for cell viability; *P < 0.05 vs. Control. Scale bar = 20 μm. (**C**) Luciferase assay showing LOX-1 as a direct target of miR-590-5p; *P < 0.05 vs. non-miR-590-5p mimic transfection group; ^†^P < 0.05 vs. miR-590-5p mimic and pmiR-LOX-1 3′UTR co-transfection group. (**D**) Proposed pathway of miR-590-5p regulating angiogenesis in endothelial cell. According to this postulate ox-LDL induces LOX-1 and inhibits miR-590-5p expression, which induces NADPH oxidase and mtROS generation resulting in pro-inflammatory activity and NF-κB p65 phosphorylation. NF-κBp65 activation results in the overexpression of VEGF, together with increased expression of pro-angiogenesis factors such as CD31, MCP-1 and IL-1β, as well as the activation of adhesion proteins VE-cadherin, p120 catenin and β-catenin. This process promotes cell migration and proliferation, and finally angiogenesis. Data based on six independent experiments.

Cell migration, as measured by the wound assay, was inhibited in the miR-590-5p mimic group and enhanced in the miR-590-5p inhibitor group (P < 0.05 vs. Control). Cell viability, as measured by the MTT assay, was also inhibited in the miR-590-5p mimic group and enhanced in the miR-590-5p inhibitor group (P < 0.05 vs. Control, Fig. 6A,B)

### LOX-1 as a direct target of miR-590-5p

As shown in Fig. 6C, luciferase activity of LOX-1 decreased when the HUVECs were transfected with the miR-590-5p mimic (100 nM) (P < 0.05 vs. Control). The miR-590-5p mimic did not decrease luciferase activity after mutating the miR-590-5p targeted site on 3′-UTR of LOX-1 (P < 0.05 vs. miR-590-5p mimic and pmiR-LOX-1 3′UTR co-transfection group).

## Discussion

In this study, we used a small concentration of ox-LDL (5 μg/mL) to mimic a model of ox-LDL-induced angiogenesis^8^,^9^. Importantly, miR-590-5p expression decreased maximally at 24 h after ox-LDL treatment when LOX-1 expression was maximally induced. Furthermore, treatment with this concentration of ox-LDL (5 μg/mL) resulted in almost doubling of VEGF expression and capillary tube formation. Notably, high concentration of ox-LDL (10 μg/mL) induced a decrease in both VEGF and capillary tube formation, perhaps because of some degree of cell death. This is consistent with the results of previous study, which indicated 10–100 μg/mL of ox-LDL are at least 1-log fold higher than those seen in normal human sera^10^.

miR-590-5p belongs to the miR-590 family, a critical group of miRNAs involved in the regulation of cell differentiation^11^. miRNAs affect target genes through degrading mRNAs or inhibiting their translation into protein^4^. A significant change in LOX-1 protein, but not mRNA, with the mimic or the inhibitor indicated that miR-590-5p inhibits LOX-1 at translational level. To confirm that LOX-1 is a direct target of miR-590-5p, we co-transfected HUVECS with miR-590-5p mimic and pmiR-LOX-1 3′-UTR or pmiR-LOX-1 mutant 3′-UTR, and the results of luciferase activity confirmed our postulate. We believe this is the first demonstration that miR-590-5p exerts an inhibitory effect on LOX-1 expression.

We studied mtROS generation in HUVECs since it is more important than cytosolic ROS generation^12^,^13^.The expression of 4 different subtypes of NADPH oxidase (p22^phox^, p47^phox^, p67^phox^, Gp91^phox^), mtROS generation and activation of p38 MAPK, ERK1/2, JNK and NF-κB p65 all were significantly (≈50%) inhibited by the transfection of HUVECs with the miR-590-5p mimic. Further, all these phenomena were markedly enhanced by the miR-590-5p inhibitor.

LOX-1 has been demonstrated to induce oxidative stress^14^, and in turn, oxidative stress stimulates LOX-1 expression in a positive feedback manner^15^. Oxidative stress accompanies atherogenesis, hypertension and several other vascular disease states. The overexpression of NADPH oxidase, mtROS generation, and activation of redox-sensitive signals [MAPKs (P38, ERK1/2 and JNK) and NF-κB p65] all are markers of LOX-1 activation^14^. There is a modest amount of oxidative stress produced in the HUVEC environment when the cells are treated with small concentrations of ox-LDL.

Ox-LDL treatment of HUVECs coated with matrigel and placement of matrigel plugs in the subcutaneously space are commonly used to check angiogenesis. In both models, we observed a robust angiogenic response. It was heartening to observe that the angiogenic response was inhibited by the miR-590-5p mimic and markedly enhanced by the miR-590-5p inhibitor. Previous studies have shown that ox-LDL stimulates expression of CD31, MCP-1 and interleukins^16^. Pro-inflammatory cytokines MCP-1 and IL-1β act as pro-angiogenic factors^17^,^18^. In keeping with these reports, our present findings demonstrate for the first time that miR-590-5p affects these angiogenic regulators.

VE-cadherin, p120 catenin and β-catenin are a group of proteins that play key roles in endothelial cell biology through control of the cohesion and organization of the intercellular junctions, and in the formation of blood vessels, regulated at least in part by LOX-1^19^,^20^. We checked the expression of these proteins in HUVECs treated with miR-590-5p mimic or the inhibitor. In keeping with our data on pro-inflammatory cytokines, we observed that the expression of these endothelial adhesion proteins to be potently regulated by miR-590-5p. These findings are in concert with our previous studies that showed an interaction between pro-inflammatory cytokines which go on to promote these adhesion molecules^20^. Along with the changes in the adhesion proteins, we observed increased cell proliferation and migration of HUVECs; and importantly, this increase was blocked by the miR-590-5p mimic and was amplified by the miR-590-5p inhibitor. It should be mentioned that adhesion proteins, VE-cadherin, p120 catenin and β-catenin, are thought of as an anti-migration mediators^21^. The increased expression of these adhesion proteins in response to ox-LDL may be a compensatory mechanism to combat inflammation. The suggested pathway of miR-590-5p regulating angiogenesis in atherosclerosis is shown in Fig. 6D.

MicroRNAs are involved in a series of diseases. Our observations of miR-590-5p regulation LOX-1 expression, and thereby the state of oxidative stress culminating in angiogenesis, indicate that miR-590-5p may be regarded as a biomarker or therapeutic strategy for angiogenesis. There are several limitations in this study: (i) we used HUVECs in this study rather than HCAECs, which were used in our previous studies^8^ and there are inherent differences in angiogenic potential of the two endothelial cell lines; (ii) our study did not identify the precise mechanism by which miR-590-5p is repressed in response to ox-LDL. We speculate that it may be due to some yet undefined signal such as long non-coding RNAs that regulate miR-590-5p expression. (iii) The effects of miR-590-5p on plaque angiogenesis should be studied in animal model of dyslipidemia (for example, Apo-E or LDLr null mice) to determine the effects of this microRNA in targeting a specific disease state.

It is possible that miR-590-5p may target proteins other than LOX-1 that influence angiogenesis. However, the focus of our study was to determine if miR-590-5p modulates LOX-1. Future studies will address regulation of other protein/s regulated by this miR that are involved in angiogenesis. MicroRNAs are widely expressed in the body, and their modulation may be more effective than the use of anti-angiogenic therapies. Therapy with miR-590-5p mimics and inhibitors may provide a useful strategy for inhibiting or promoting angiogenesis.

## Methods

### Cell culture, treatments and miRNA transfection

Vascular cell basal medium was used to culture primary HUVECs (ATCC PCS-100-013, Beijing, China), which included bovine brain extract (BBE) (2%), rh EGF (5 ng/mL), L-glutamine (10 mM), heparin sulfate (0.75 Units/mL), hydrocortisone hemisuccinate (1 μg/mL), fetal bovine serum (2%) and ascorbic acid (50 μg/mL). Cells were seeded onto six-well plate at a density of 4 × 10^5^ cells per well and incubated in a humidified atmosphere with 5% CO_2_ at 37 °C, before transfected with syn-hsa-miR-590-5p miScript miRNA mimic or anti-hsa-miR-590-5p miScript miRNA inhibitor (custom products from QIAGEN, Shanghai, China). A scrambled random sequence was used as negative control for both mimics and inhibitors to eliminate non-specific effect of miRs on LOX-1 expression (custom products from QIAGEN, Shanghai, China). Lipofectamine 2000 (Invitrogen Corp, Shanghai, China) was used for transfection. A > 60% transfection efficiency was to determine that the mimics or inhibitors can be used in HUVECs. After 6 h of transfection, fresh medium was added to the wells and HUVECs were incubated for additional 24 h before subsequent experiments. For induction of angiogenesis, cells were then incubated with different concentrations (0, 1, 5, 10 μg/mL) of ox-LDL (TBARs 90 nmol MDA/mg protein, Alfa Aesar, Beijing, China) for 24 h. Since 5 μg/mL concentartion of ox-LDL resulted in a consistent and significant angiogenesis, this concentration was used in subsequent experiments.

### Matrigel plug implantation and treatment with ago- and antago- miR-590-5p

Eight to ten week old C57BL/6 wild type mice were implanted with matrigel basement matrix plug (BD Biosciences, Shanghai, China) containing HUVECs subcutaneously on day 0 followed by tail-vein injections of saline, AgomiR-590-5p or AntagomiR-590-5p (10 mg per kg body weight in 0.2 ml volume) on days 1, 3 and 5. AgomiR-590-5p or AntagomiR-590-5p was obtained from RiboBio. Co, Guangzhou, China. Mice were sacrificed on Day 6, and matrigel plugs harvested, and embedded in paraffin and sectioned. Hematoxylin and eosin (H&E staining) and CD31 immunofluorescence were performed following the standard protocol. The sequence of AgomiR-mmu-590-5p was: 5′-CAGGCCGAUUGCGAUGCAAUA-3′, and that of AntagomiR-mmu-590-5p was 5′-AAAUAUGCUGUAUGUCAUGUGUU-3′. All experimental procedures were performed in accordance with protocols approved by the Institutional Animal Care and Usage Committee of Anhui Medical University, and conformed to the Guidelines for the Care and Use of Laboratory Animals published by the National Institutes of Health.

### Measurement of mitochondrial reactive oxygen species

Intracellular mitochondrial ROS was assessed using MitoSOX™ Red mitochondrial superoxide indicator (Invitrogen Corp, Shanghai, China). Flow cytometery was used to quantify ROS generation, and the data were quantified by WinMDI2.9 software as described previously^13^.

### Capillary tube formation

After thawing on ice overnight, Matrigel (BD Biosciences, Shanghai, China) was spread over each well. Before seeding HUVECs, plates were incubated at 37 °C for 1.5 h, and cells were transfected with miR-590-5p mimic or the inhibitor, and incubated in medium with 10% FBS with ox-LDL for another 48 h. After washing, plates were fixed by 70% ice-cold ethanol. Cells were then observed under microscopy for visualizing capillary formation as described previously^10^. Since FBS may influence capillary tube formation, we compared the effect of FBS by studying capillary tube formation in medium with or without FBS, and did not see any significant difference (Fig. S2).

### MTT assay

Cell viability was measured using the MTT assay kit (Sigma). Briefly, 48 h after transfection, 0.5 mg/ml MTT was added to each well and cells were incubated for additional 6 h at 37 °C. After wash with PBS, absorbance was measured at 550 nm.

### Wound healing assay

HUVECs were cultured on gelatin-coated, 9 cm^2^ dishes until confluence. A razor blade was used to wound monolayers, and cells were treated with 5 μg/mL ox-LDL (plus solvent, miR-590-5p mimic or the inhibitor). Migration of cells was analyzed 24 h later. Results were calculated as number of migrated cells, mean ± SD of three independent experiments each in triplicate.

### Luciferase assay report

LOX-1 as a direct target of miR-590-5p was confirmed by luciferase assay report. Human LOX-1 mRNA 3′-UTR and human LOX-1 mRNA mutant 3′-UTR reporters were synthesized following a previously described protocol^22^.

### Quantitative real-time RT-PCR

Real-time RT-PCR was performed as described previously^23^. Briefly, total RNA was extracted using TRIZOL reagent. cDNA (200 ng) from reverse transcription was amplified using 300 nM primers (B&M Biotech Co, Beijing, China). Primer sequences are shown in Table 1. GoTaq® qPCR Master Mix kit was used to detect each mRNA. Applied Biosystems 7900 was used for performing real-time PCR. Cycling condition and reaction system were followed as per standard protocol. GAPDH and U6 genes were used for normalizing the comparative threshold cycles values.

### Western blotting

Protein extraction and western blot were performed as described previously^10^. SDS-PAGE (10%) was used to separate protein aliquots (35 μg). Image J software was used to quantify and analyze intensities of the bands. Antibodies were purchased from Abcam (Hongkong, China): (LOX-1, p38 MAPK, CD31, IL-1β, MCP-1, p-VE-cadherin, p-p120 catenin and p-β-catenin), Santa Cruz Biotechology (Shanghai, China): (VEGF, ERK1/2, JNK, p-JNK (Thr 183/Tyr 185), NF-κB p65 and β-actin; Cell signaling (Shanghai, China): (p-ERK1/2(Thr202/Tyr204), p-NF-κB p65(Ser536), p-p38 MAPK (Thr180/Tyr182)).

### Statistical analysis

All studies were performed at least 5 different times. Two-tailed t test or single-way ANOVA (multiple means) were used to analyze the data. Results are presented as mean ± SD. A *P* value < 0.05 was considered significant.

## Additional Information

**How to cite this article**: Dai, Y. *et al*. MiR-590-5p Inhibits Oxidized- LDL Induced Angiogenesis by Targeting LOX-1. *Sci. Rep.* **6**, 22607; doi: 10.1038/srep22607 (2016).

## Supplementary Material

Supplementary Information

## Figures and Tables

**Table 1:** Primer sequences for real-time PCR.

Primer Sequences	Forward Primer	Reverse Primer
*LOX-1*	5′-CTGGATTGGATTGCATCGGAA-3′	5′-CAGCTCCGTCTTGAAGGTATG-3′
*p22*^*phox*^	5′-TGCCAGTGTGATCTATCTGCT-3′	5′-TCGGCTTCTTTCGGACCTCT-3′
*p47*^*phox*^	5′- ACACCTTCATTCGCCATATTGC-3′	5′- TCGGTGAATTTTCTGTAGACCAC-3′
*P67*^*phox*^	5′- GAGGGATGCTCTACTACCAGAC-3′	5′- CCTCGAAGCTGAATCAAGGC-3′
*Gp91*^*phox*^	5′-GACGCTGCTGTTTGAGAAATG-3′	5′-ATCGCTGAAGAAGGGTTTGTG-3′
*CD31*	5′-AACAGTGTTGACATGAAGAGCC-3′	5′-TGTAAAACAGCACGTCATCCTT-3′
*MCP-1*	5′- CAGCCAGATGCAATCAATGCC-3′	5′- TGGAATCCTGAACCCACTTCT-3′
*IL-1β*	5′- ATGATGGCTTATTACAGTGGCAA-3′	5′- GTCGGAGATTCGTAGCTGGA-3′
*VEGF-α*	5′-AGGGCAGAATCATCACGAAGT-3′	5′-GCTGCGCTGATAGACATCCA-3′
*GAPDH*	5′- AGGTCGGTGTGAACGGATTTG-3′	5′- TGTAGACCATGTAGTTGAGGTCA-3′
*has-miR-590-5p*	5′-GGAATTCTTCAGTTGTAACCCAG-3′	5′-CGGGATCCTTGAGATGTCACCAA-3′
*U6*	5′-CTCGCTTCGGCAGCACA-3′	5′-AACGCTTCACGAATTTGCGT-3′
