# Supplementary material for: MiR-590-5p Inhibits Oxidized- LDL Induced Angiogenesis by Targeting LOX-1
Source: Sci Rep. 2016 Mar 2;6:22607. doi: 10.1038/srep22607 (PMC4773867; doi:10.1038/srep22607)
Supplement: Supplementary Information [file srep22607-s1.pdf]

MiR-590-5p Inhibits Oxidized- LDL Induced Angiogenesis by Targeting LOX-1

Yao Dai[1,2,3], Zhigao Zhang[3],Yongxiang Cao[3], Jawahar L. Mehta[2*], Jun Li [1*]

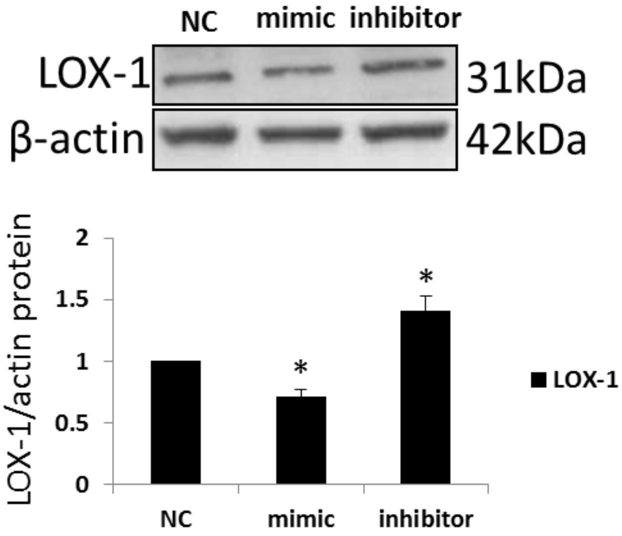

Figure Legend
S1. Effect of miR-590-5p mimic (100nM) and inhibitor (200nM) on LOX-1 protein expression compared to negative control. *P<0.05 vs. negative control; NC, negative control; other abbreviations as in previous figures; Data based on 3 experiments.

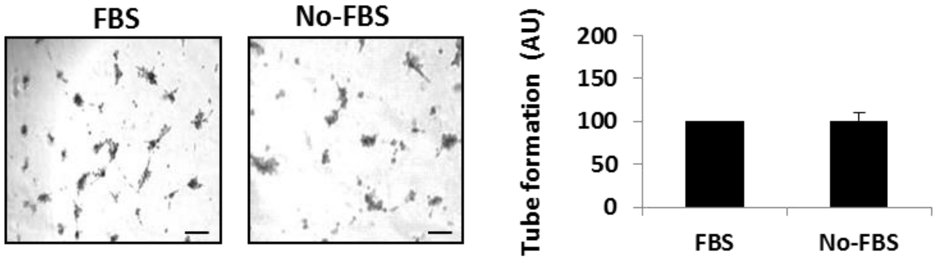

Figure Legend
S2. Capillary tube formation in media with or without fetal bovine serum (FBS).
